# Effects of Beetroot Juice Ingestion on Physical Performance in Highly Competitive Tennis Players

**DOI:** 10.3390/nu12020584

**Published:** 2020-02-23

**Authors:** Álvaro López-Samanes, Alberto Pérez-López, Victor Moreno-Pérez, Fabio Yuzo Nakamura, Jorge Acebes-Sánchez, Iñaki Quintana-Milla, Antonio J. Sánchez-Oliver, Diego Moreno-Pérez, Valentín Emilio Fernández-Elías, Raúl Domínguez

**Affiliations:** 1 School of Physiotherapy, Faculty of Health Sciences, Universidad Francisco de Vitoria, 28223 Madrid, Spain; 2 Department of Biomedical Sciences, Area of Sport and Physical Education, Faculty of Medicine and Health Sciences, University of Alcalá, 28805 Madrid, Spain; alberto_perez-lopez@hotmail.com; 3 Center for Translational Research in Physiotherapy, Department of Pathology and Surgery, Universidad Miguel Hernández, Elche, San Juan, 03202 Alicante, Spain; vmoreno@goumh.es; 4 Associate Graduate Program in Physical Education UPE/UFPB, 58051-970 João Pessoa, PB, Brazil; fabioy_nakamura@yahoo.com.br; 5 Exercise and Sport Sciences, Faculty of Health Sciences, Universidad Francisco de Vitoria, 28223 Madrid, Spain; j.acebes.prof@ufv.es (J.A.-S.); i.quintana.prof@ufv.es (I.Q.-M.); 6 Departamento de Motricidad Humana y Rendimiento Deportivo, Universidad de Sevilla, 41013 Sevilla, Spain; sanchezoliver@us.es; 7 Department of Education, Research and Evaluation Methods, Universidad Pontifica de Comillas, 28015 Madrid, Spain; dmperez@comillas.edu; 8 Faculty of Sports Sciences, Universidad Europea de Madrid, 28670 Madrid, Spain; valentin.fernandez@universidadeuropea.es; 9 College of Health Sciences, Isabel I University, 09003 Burgos, Spain; raul_dominguez_herrera@hotmail.com

**Keywords:** NO precursors, racket sports, intermittent sports, ergogenic aid

## Abstract

Beetroot juice (BJ) contains high levels of inorganic nitrate (NO_3_^−^) and its intake has good evidence in increasing blood nitrate/nitrite concentrations. The ingestion of BJ has been associated with improvements in physical performance of endurance sports, however the literature in intermittent sports is scarce. The aim of this study was to investigate whether BJ could improve physical performance in tennis players. Thirteen well-trained tennis players (25.4 ± 5.1 years) participated in the study during their preparatory period for the tennis season. Subjects were randomly divided into two groups and performed a neuromuscular test battery after either BJ or placebo (PLA) consumption. Both trials were executed on two separate days, in randomized order, with one week of wash out period. The test battery consisted of serve velocity test (SVT), countermovement jump (CMJ), isometric handgrip strength (IHS), 5-0-5 agility test (5-0-5), and 10 m sprint (10-m). No significant differences were found in SVT (1.19%; *p* = 0.536), CMJ (0.96%; *p* = 0.327), IHS (4.06%; *p* = 0.069), 5-0-5 dominant and nondominant side (1.11–2.02%; *p* = 0.071–0.191) and 10-m (1.05%; *p* = 0.277) when comparing BJ and PLA ingestion. Thus, our data suggest that low doses of BJ (70 mL) consumption do not enhance tennis physical performance.

## 1. Introduction

Tennis is an intermittent sport characterized by high-intensity efforts, such as accelerations, decelerations, running sprints, and frequent changes-of-direction (COD) [1], interspersed with periods of low-to-moderate intensity or rest (short breaks between points (10–20 s) and longer rest periods between games and sets (90–120 s)), with match duration between 1 and 5 h [2,3]. Tennis success has been related to a mixture of technical, tactical, psychological, and physical components [4]. High performance in tennis is associated with higher values of strength and power output [5], agility [6], and serve velocity [7]. Other secondary determinant aspects have been proposed, such as environmental [8], nutrition [9], chronobiological aspects [10], and recovery strategies [11]. 

Due to the explosive demands that characterize tennis competition, identification of nutritional supplements and ergogenic aids that might contribute to enhance physical performance during training/matches could be an useful strategy to be adopted by elite tennis players [12]. However, despite the popularity of tennis around the world, studies developed on the effects of nutritional supplements with the objective of acutely enhancing tennis performance are scarce, reduced to a few studies on some ergogenic aids such as caffeine [13,14], creatine [15,16], sodium bicarbonate [17], or sodium citrate [18].

Beetroot juice (BJ), rich in inorganic nitrate (NO_3_^−^), is considered to be an ergogenic aid as it may serve as a precursor of nitric oxide (NO) through the nitrate-nitrite-NO pathway [19]. Briefly, although NO_3_^−^ is considered biologically inert, after BJ ingestion this molecule is reduced to nitrite (NO_2_^−^) and subsequently to NO [20]. After absorption in the oral cavity or the intestine, circulating NO_2_^−^ serves as a substrate for O_2_^−^ independent of NO generation, a process potentiated by hypoxia and acidosis conditions [20]. An increased NO presence derived from NO_3_^−^ supplementation improves vasodilation, increasing blood flow to the muscles [21,22,23], and facilitates force production in vivo, reducing ATP cost in exercised muscles [24,25]. Hence, BJ supplementation has showed the capacity to improve endurance performance [26] and accelerate post-repeated-sprint recovery [27]. Furthermore, it has been suggested that ingestion of BJ may be particularly effective in intermittent sports at augmenting different physiological processes, such as calcium-handling proteins and contractile force in type II (fast-twitch) muscle fibers [28]. However, controversial findings are reported in the scientific literature, with some studies showing improvements in repeated-sprint performance by reducing time to reach peak power (~2.8) [29,30], while other studies do not report any difference between BJ and placebo (PLA) conditions [31]. In tennis, only one study developed by Aksit et al. [32] examined the relationships between simulated tennis performance test and circulating NOx (i.e., sum NO_3_^−^ + NO_2_^−^) levels. However, no study has evaluated the effect of BJ ingestion in tennis. Thus, the aim of this study was to analyze the effect of BJ ingestion on physical tennis performance.

## 2. Materials and Methods

### 2.1. Subjects

Thirteen highly competitive male tennis players (age, 25.4 ± 5.1 years; body mass, 74.7 ± 8.8 kg; height, 1.82 ± 0.1 m; body mass index (BMI), 22.6 ± 1.4; tennis experience, 14.9 ± 7.4 years; hours training/week, 12.2 ± 3.1 h/week) were recruited to participate in the study. Four tennis players had an ATP ranking (i.e., professional tennis ranking) between 650 and 1800, and nine were among the 350 best Spanish senior tennis players. After being fully informed of the experimental protocols, participants gave their informed written consent to participate. The Bioethics Commission of the University (number 46/2018) approved the study, which complied with the recommendations of the Declaration of Helsinki.

### 2.2. Experimental Design

**Figure 1:**
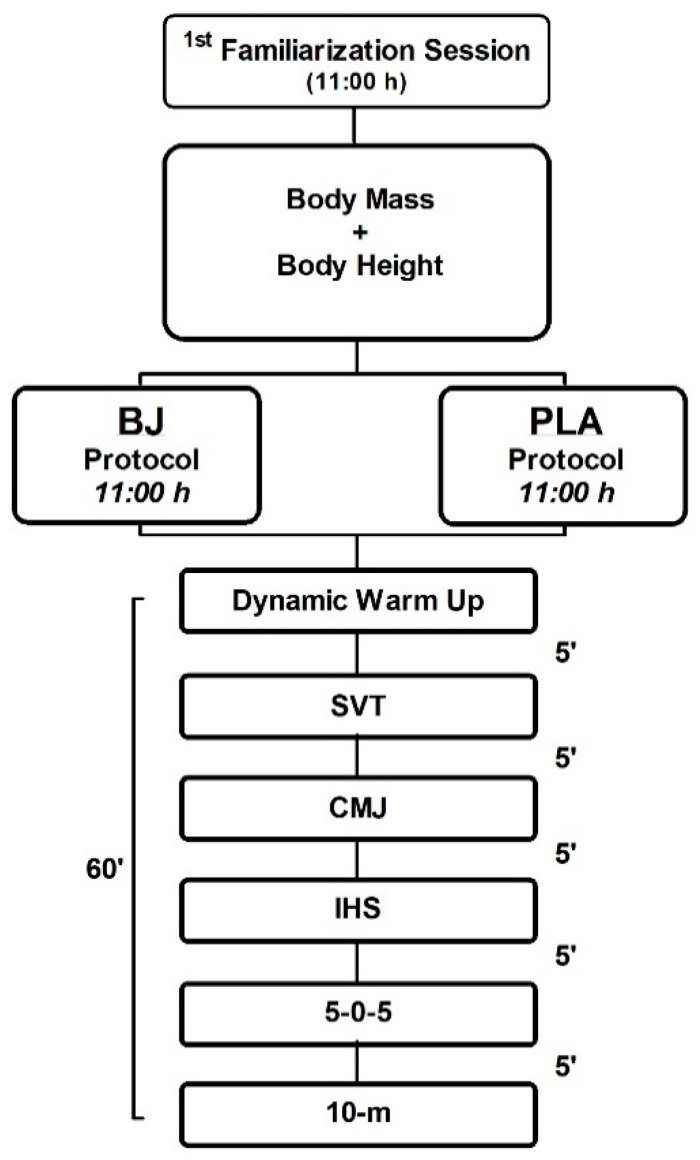
Experimental design flow chart. Abbreviations: BJ: beetroot juice; PLA: placebo; SVT: Serve velocity test; CMJ: counter movement jump; IHS: isometric handgrip strength; 5-0-5: Agility Test: 5-0-5; 10-m: 10 m sprint.

The study design was randomized cross-over, placebo-controlled, and double-blind. Participants arrived at the training facilities on two separate days under the same experimental conditions with 1 week between protocols to allow recovery and substance wash out. Participants were instructed to avoid any form of exercise leading up to each test and the ingestion of caffeine/alcohol in the 24 h prior to the testing. In session 1, participants were subjected to a preliminary assessment of body composition and underwent a familiarization session of the neuromuscular test battery. Then, on two separate occasions (sessions 2 and 3), when they arrived at the tennis training facilities, participants were provided with a supplement containing 70 mL of either BJ (Beet IT; James White Drinks Ltd., Ipswich, UK) or PLA (ECO Saludviva, Alicante, Spain). The trial was double-blinded such that one researcher (D.M.-L.) allocated all the participants’ drinks in a counter-balanced fashion (in each trial, 50% of participants ingested PLA and 50% ingested BJ beverages) with random assignment to each supplement (Research Randomizer, www.randomizer.org). Three hours after intake of BJ or PLA, subjects performed a standardized dynamic warm up protocol [33], and then a neuromuscular test battery was carried out, consisting of a tennis serve velocity test (SVT), countermovement jump (CMJ), isometric handgrip strength (IHS), 5-0-5 agility test (5-0-5), and 10 m sprint (10-m) (Figure 1). Furthermore, in both trials, the experimental procedures were performed at the same hour of the day to avoid the influence of circadian rhythms on performance [10].

### 2.3. Beetroot Juice vs. Placebo Ingestion

In addition, the required sample size was determined by statistic power calculation on the basis of previous studies [27]. The minimum number of participants required to detect an 8% ± 6% difference in counter movement jump (CMJ) performance between two groups, with a power of 0.80 and two-tailed α level set at 0.05, was estimated as seven per group using the sample size package G Power 3.1 (Kiel, Germany). After an overnight fast, participants arrived to the laboratory 3 h before undertaking the neuromuscular test battery. Upon arrival, they were provided with a drink containing either 70 mL of BJ (containing 6.4 mmol of NO_3_^−^) (Beet-It-Pro Elite Shot, James White Drinks Ltd., Ipswich, UK) or PLA (0.04 mmol of NO_3_^−^), as described elsewhere [34]. All participants were instructed to follow a diet sheet the day before each trial that consisted of 60% carbohydrates, 30% fat, and 10% proteins. Dietary NO_3_^−^ was limited by providing subjects with a list of NO_3_^−-^rich foods (e.g., beetroot, celery, or spinach), which they should avoid in the 48 h before each trial. In addition, in the 24 h leading up to each test, subjects were encouraged to avoid brushing their teeth or using any oral antiseptic rinse, or ingesting gum, sweets, stimulants (e.g., caffeine), or alcohol that could alter the oral microbiota and interfere with NO_3_^−^ reduction. At the end of both trials, participants were asked to guess the order of the trials to determine whether they have identified in which day they ingested BJ and PLA.

### 2.4. Environmental Conditions and Rate of Perceived Exertion (RPE)

During the whole duration of each testing session, air temperature and humidity were measured with a portable weather station (WMR 108, Mextech, India). Data were averaged to obtain the mean morning and afternoon temperature (°C) and relative humidity (%). The perceptual training intensity for each subject during the intervention was registered using the rating of perceived exertion (RPE) within 30 min of the warm up termination.

### 2.5. SVT

Serve speed was measured by a radar gun (model Pocket Radar Ball Coach PR1000BC, Republic of South Korea), which was set on “continuous mode” to detect maximal ball speed (40 to 210 km/h range). Calibration was performed according to the manufacturer’s specifications prior to each test. The serve test procedure was conducted as previously described [35]. Briefly, the radar was positioned in the tennis court in the center of the baseline, 4 m behind the server, aligned with the approximate height of ball contact (~2.2 m) and pointing down the center of the court. After a brief warm up consisting of dynamic movements of the shoulder, five-second services, and five submaximal services, the serve velocity test was performed. Participants were required to serve in a 1 × 1 m^2^ area allocated in the farther diagonal corner of the serving area, performing five maximal speed serves in as few attempts as possible. The mean velocity of the five serves was calculated for further analysis.

### 2.6. CMJ

Participants completed three repetitions of a CMJ using an infrared jump system (Optojump, Microgate, Italy) according to standard methodology [36]. Each participant performed three maximal CMJ interspersed with 45 s of passive recovery. The highest height out of the three jumps was recorded.

### 2.7. IHS

Two maximum isometric voluntary contractions were measured in the dominant hand using a calibrated handgrip dynamometer (Takei 5101, Tokyo, Japan). Volunteers sat with 0 degrees of shoulder flexion, 0 degrees of elbow flexion, and the forearm and hand in a neutral position [10]. The highest value out of two attempts was recorded as the maximum voluntary handgrip strength.

### 2.8. 5-0-5

The athletes’ ability to perform a single, rapid 180° change of direction over a 5 m distance was measured (Smartspeed, Fusion Sport, Australia) using a modified version (stationary start) of the 5-0-5 agility test. Players started with their preferred foot behind the starting position (dominant (DOM) or nondominant side (NO-DOM)) and accelerated voluntarily, sprinting with maximal effort without a racquet [37]. Each repetition was initiated from a standing position, 50 cm behind the photocell gate which started a digital timer. Two trials were completed, one each pivoting the left and right feet, and the highest value out of the two attempts was recorded for subsequent analysis.

### 2.9. 10-m

The time during a 10 m dash in a straight line was measured using two photocell gates placed 1 m above the ground level (Smartspeed, Fusion Sport, Australia). Each sprint was initiated from a standing position, 50 cm behind the photocell gate which started a digital timer. Another photocell gate was placed at the finish line to stop the timer [10]. The best performance out of two repetitions, separated by 1 min recovery period, was recorded for subsequent analysis.

### 2.10. Statistical Analysis 

Data are presented as means and standard deviation. The Shapiro–Wilk test was used to assess the distribution of data. All variables were compared using the Student’s t-test for related variables (BJ vs. PLA). The significance level was set at *p* ≤ 0.05. Cohen’s d formula for effect size (ES) was used, and the results were based on the following criteria: trivial (0–0.19), small (0.20–0.49), medium (0.50–0.79), and large (0.80 and greater) [38]. All the statistical analyses were performed using SPSS software, version 22.0 (SPSS Inc., Chicago, IL, USA).

## 3. Results

### 3.1. Enviromental Conditions and RPE

Ambient condition data were averaged to obtain the mean morning temperature (11.0 ± 3.4 °C, *p* = 0.383) and relative humidity (36% ± 5%, *p* = 0.792). Regarding RPE values, no differences were founded between BJ and PLA conditions (3.8 vs. 4.2, *p* = 0.248). In addition, the order of the experimental trials was correctly identified by 53.8% of the participants (7 of 13). 

### 3.2. SVT, CMJ, IHS, 10-m, and 5-0-5 Tests

**Figure 2:**
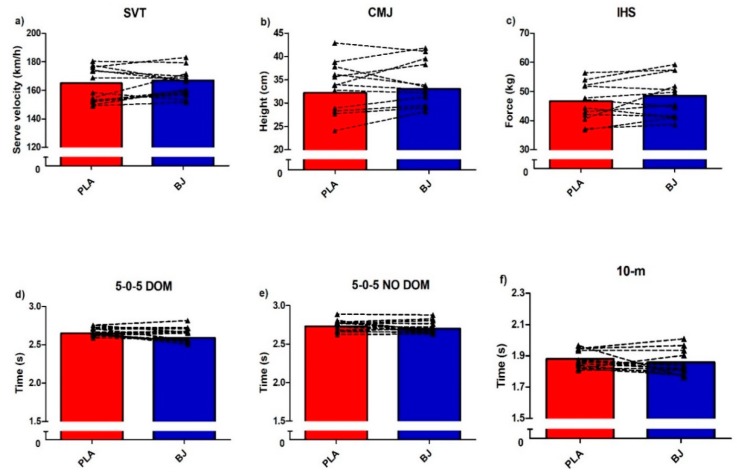
Effects of beetroot juice (BJ) or placebo (PLA) on (**a**) SVA: serve velocity test; (**b**) CMJ: countermovement vertical jump height; (**c**) IHS: isometric handgrip strength; (**d**) 5-0-5 DOM: agility test dominant side; (**e**) 5-0-5 NO-DOM: agility test nondominant side; (**f**) 10-m: 10 m sprint.

No significant differences were founded between conditions (BJ vs. PLA) in any of the physical performance tests analyzed, such as SVT (*p* = 0.536; ES = 0.16; Figure 2a), CMJ height (*p* = 0.327; ES = −0.14; Figure 2b), IHS test (*p* = 0.069; ES = 0.26; Figure 2c), 5-0-5 DOM (*p* = 0.071; ES = 0.69; Figure 2d), 5-0-5 NO-DOM (*p* = 0.191; ES = −0.38; Figure 2e), and 10-m test (*p* = 0.277; ES = 0.39; Figure 2f).

## 4. Discussion

The ergogenicity of BJ has been evidenced in some intermittent sports (e.g., soccer, rugby, hockey) [39,40,41], however, other sports such as basketball showed no benefits with BJ ingestion [42]. To our knowledge, no previous studies have evaluated its efficacy on tennis physical performance. Thus, there is a need to understand how BJ can impact physical performance of tennis players to ascertain if the ingestion of BJ might be beneficial. In comparison with PLA values, BJ ingestion showed no statistical differences in the performance variables analyzed, such as SVT (1.2%), IHS (4.1%), 10-m (−1.1%) and 5-0-5 in the DOM (−2.0%) and NO-DOM side (−1.1%). Overall, the results indicate that BJ in a dose of 70 mL does not produce an improvement of physical tennis performance in highly trained tennis players, that is in agreement with previous studies in other intermittent team sports (e.g., basketball), where no significant differences were found in agility, sprint, isometric strength, or even match play demands values [42].

Beetroot juice consumption promotes an enhanced endurance performance in several populations of athletes [26], potentially due to improved vasodilatation, which facilitates an increase in blood flow to the exercised muscles [22,23] and stimulates a reduction in the O_2_ needed during submaximal efforts [25]. However, in intermittent efforts, there is controversial evidence regarding the ergogenic effect of BJ [40,42]. While several days of BJ administration may stimulate a modification of contractile properties of the muscle, allowing the generation of higher rates of power, enhancing speed in short distances and duration tests [27,29,30], no significant differences were found after the acute ingestion of BJ [28,36]. In this study, we observed no changes in the different explosive actions related to physical tennis performance (e.g., SVT, CMJ, HIS, 5-0-5, and 10-m) with the ingestion of BJ against PLA.

Therefore, in this study, the acute ingestion of low doses of NO_3_^−^ (6.4 mmol) did not stimulate an increase in physical performance in tennis. These results are opposed to the evidence provided by Coggan et al. [43], who found an increased muscle speed and power in healthy men and women after isokinetic knee extension performed at high velocity (360 degree/s), a task where type II muscle fibers are highly recruited and expected to generate elevated force and power production. However, the NO_3_ dose provided in both studies was substantially different (6.4 vs. 11.2 mmol) and, thus, low doses of NO_3_ may not be enough to cause an improvement of contractile properties of the muscle, potentially due to insufficient activation of cyclic guanosine monophosphate (cGMP) [43]. 

The tennis serve is a technical/tactical skill which plays a critical role in the outcome of a tennis match, and, particularly, ball velocity is a key variable in determining a successful play [44]. We observed that serve velocity increased 1.2% with BJ ingestion, not reaching statistical significance in our study (*p* = 0.536). Although no previous studies have been developed with the ingestion of BJ on physical tennis performance, the ingestion of other ergogenic aids or nutritional supplements such as caffeine (3.77% improvement against PLA) in tennis serve [13] or sports drinks reducing fatigue after tennis match [45] have demonstrated higher effects in tennis performance compared with BJ ingestion. Also, vertical jumps are commonly involved during tennis training/matches in different tennis-specific actions (e.g., serves/smashes). According to previous literature, the ingestion of BJ in small doses (70 mL) does not improve jump height (2.3% improvement without reaching statistical significance) [46]. These results are in agreement with the data obtained in our study (*p* = 0.327). The differences found in both studies could be attributed to the differences in the sample characteristics. In our study, we studied highly trained male tennis players, while the study of Cuenca et al. [46] was conducted with young active male players. In addition to the evidence previously reported, some studies did not find a positive effect of NO_3_^−^ supplementation on peak power during a cycle ergometer test [29,47], while other studies found improvement in peak power on a cycle ergometer [46,48,49] and strength performance [50,51], but not during a vertical jump [46]. Hence, controversial evidence about the effects of BJ supplementation on several aspects of physical performance, such as muscle force or power, are presented in the literature.

In comparison with PLA, BJ ingestion did not stimulate an increased IHS on the dominant hand, (4.1%, *p* = 0.069). Our data are in agreement with a previous study published by Clifford et al. [25] which showed no statistical differences in maximal isometric voluntary contractions (1.86%) after ingestion of BJ in different team sport athletes (e.g., soccer, rugby, etc.) Although IHS is not a specific action of tennis match play, this test constitutes a simple method to test the effect of BJ on force production and might be an indication of higher force capacity. In addition, Girard et al. [5] established that isometric handgrip strength values are related to higher physical performance in adolescent tennis players. However, the acute administration of BJ did not cause a significant effect on this test.

During tennis performance, the ability to change direction on the tennis court has been related to tennis performance since during a tennis point, players complete an average of four directional changes per point, reaching up to 15 directional changes during long rallies [52]. In fact, according to Roetert et al. [6], agility could be the only physical performance variable predictive of competitive rankings in young male tennis players. In our study, no statistically significant differences were found after BJ ingestion in the DOM (−2.02%) and NO-DOM side (−1.11%), compared with PLA ingestion agility values (*p* = 0.071). In conjunction with the ability of change-of-direction on the tennis court, the capacity to cover short distances (i.e., <20 m) in less time is also critical for tennis performance and has been related to higher tennis physical performance. According to previous literature, controversial findings have been reported regarding the ergogenic effect of BJ on sprint performance. While some investigations reported benefits from BJ ingestion (140 mL) [40], other studies did not find an effect [42,53]. To our knowledge, this is the first study to analyze the effects of BJ on agility in tennis. 

## 5. Limitations

BJ dose (70 mL) used during this study was insufficient to stimulate an ergogenic effect. While some studies have reported improvements with this dose in high-intensity efforts [34,46], one study found that a dose of 4.2 mmol of NO_3_^−^ (70 mL) was insufficient to improve the time to task failure; however the authors observed increments with doses between 8.4 mmol of NO_3_^−^ (140 mL) and 16.8 mmol of NO_3_^−^ (280 mL) [30]. Further studies in which a larger sample size will be recruited are required to corroborate the results obtained in this study (*n* = 13). In addition, other limitations of our study were that NO_2_^−^ and NO_3_^−^ plasma concentrations were not measured and the authors have limited ability to alter the diet in these athletes, potentially compromising their dietary restriction of nitrate-containing foods. Thus, despite the fact that our research reported no ergogenic effects after the ingestion of BJ, higher doses of BJ may promote an ergogenic effect, and, thus, future studies should examine the effects of higher doses of NO precursors than the one used in the current study.

## 6. Conclusions

In summary, the results of this study indicate that the ingestion of 70 mL of BJ (4.2 mmol) does not improve serve velocity, jump height, isometric handgrip strength, agility performance, and sprint speed in highly trained tennis players. Therefore, low doses of NO precursors may not stimulate ergogenic effects on tennis physical performance.
